# Process and analytical strategies for the safe production of mRNA vaccines and therapeutics

**DOI:** 10.1007/s11033-026-11455-0

**Published:** 2026-01-20

**Authors:** Cinderella J. A. Nowak, Sha Liu, Robert J. Falconer, Lukas Gerstweiler

**Affiliations:** School of Chemical Engineering, Faculty of Sciences, Engineering and Technology, Adelaide University, Adelaide, SA 5005 Australia

**Keywords:** MRNA vaccines/Therapeutics, Impurities, In vitro transcription, Critical quality attributes, Bio-manufacturing, High-throughput, HPLC

## Abstract

The production of high-purity mRNA drug substances by in vitro transcription utilizing T7 RNA polymerase can be challenging due to the formation of product related impurities. These include double-stranded RNA, fragmented mRNA, and uncapped transcripts. This review examines the known mechanisms underlying the formation of the major mRNA impurities during in vitro transcription (IVT), their biological impact and strategies for their mitigation. Some companies have utilised engineered T7 RNA polymerases and optimized transcription conditions to improve mRNA purity. There is a growing focus on refining upstream and downstream processes to improve mRNA purity. The current analytical approaches for impurity detection and quantification are reviewed. These range from immunological assays to advanced chromatographic and sequencing technologies. Continued innovation is needed to develop the next generation of high-throughput, cost-efficient analytical methods for quantifying mRNA impurities. Together improved transcription, purification and analysis enable the manufacture of safe efficacious mRNA for vaccines and therapeutics.

## Introduction

mRNA therapies and vaccines offer a promising addition to conventional medicines due to their rapid, scalable manufacturing, precise, sequence-defined control over protein expression, and potential to treat a wide range of diseases, including infectious diseases, cancers, and genetic disorders [1–3]. The earliest clinical trial for an mRNA cancer vaccine was conducted in 1999 using mRNA-engineered dendritic cells (NCT00004211) followed by the first clinical trial in which mRNA was directly injected into the humane body in 2004 (NCT00204607) [4–6]. However, mRNA technology has only recently become a major focus of vaccine research, particularly since the COVID-19 pandemic ushered in a new era of vaccines against viral diseases. mRNA therapeutics also have the potential to be used as protein-replacement medicines and for immune therapies for the treatment of cancer [7].

mRNA therapeutics production can be inexpensive and easily scaled to mass production. The manufacturing cost for the COVID-19 vaccine by Moderna and Pfizer is estimated to $1–3 per dose and was used to vaccinate billions of people around the world during the COVID pandemic [8, 9].

Commercial mRNA is typically produced by in vitro transcription (IVT) using a DNA template encoding the desired sequence and an RNA polymerases [3]. While this process efficiently generates the desired single-stranded mRNA (ssRNA), it also results in undesirable by-products including mRNA aggregates, abortive transcripts and immunostimulatory double-stranded RNA (dsRNA), which formation during IVT is not well understood [10–12].

Several studies have further investigated the mRNA impurities’ impact on immunological responses and have shown that dsRNAs are innate immune response-activators capable of triggering the cytosolic sensors RIG-I and MDA-5, leading to inflammatory side-effects in the patient’s body [13–15].

As a result, the ribosomal protein translation can be inhibited by dsRNA, leading to a 10- to 1000-fold decrease in target protein production [16].

The immunogenicity of mRNA can be reduced by incorporating modified nucleotides, which reduce unwanted inflammatory reactions and increase the translation of desired proteins [17]. Other impurities that can occur during the IVT process are RNA-DNA hybrids, which can be detected by the pattern recognition receptors (PRRs) TLR9, NLRP3 and cGAS and hence can activate innate immune signaling pathways [18–20].

To produce mRNA therapeutics with low innate immune stimulation, additional downstream purification steps, such as reverse phase HPLC (RP-HPLC), are necessary to remove immunogenic product related impurities, such as dsRNA [21–23]. Apart from the immunogenic by-products it is necessary to remove other impurities like abortive transcripts, aggregates, residual DNA template and T7 enzyme as they can decrease the mRNA purity and hence efficiency [24]. Removing product related impurities is challenging due to similar size and characteristics to the product. This can lead to high losses during downstream purification, decreasing the yield and ultimately increasing manufacturing costs.

There is published guidance for manufacturers of mRNA vaccines and therapeutics. The U.S. Pharmacopeia (USP) has published draft guidelines on the analytical procedures for measuring mRNA quality [25]. This was written for mRNA manufacturers, analytical laboratories and the regulatory agencies. It provides a list of the analytical methods for the common quality attributes of mRNA vaccines. Manufacturers should supplement the analytical methods in the USP guidelines, with methods to measure mRNA quality attributes identified by their in-house risk-based quality assessment of the specific mRNA therapeutic or vaccine product, and its manufacturing process [26].

Currently, there are no specific thresholds written in standardized guidelines for mRNA impurities, but some recommend a dsRNA content of less than 0.5% of the product mass [27]. Quantitative limits for other impurities such as abortive transcripts and RNA-DNA hybrids are still lacking.

In this review, we discuss IVT impurities and process-related impurities, as well as their mitigation strategies. Further, the current analytical methods for mRNA purity and integrity used during the manufacturing process will be compared and their relative advantages and limitations will be discussed. Finally, we highlight challenges and future directions for mRNA manufacturing, including emerging regulatory guidelines and AI/ML-driven tools.

## General structure and manufacturing pathway of mRNA

**Fig. 1:**
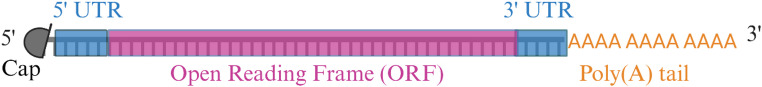
General structure of an mRNA. It consits an ORF flanked by 5’ and 3’ UTR’s, a 5’ cap and a 3’ ploy(A) tail. Created in BioRender. Nowak, C. (2025) https://BioRender.com/ih33dyc

The general structure of eukaryotic mRNA has a 5’ Cap, a 3’ poly(A) tail and an open reading frame (ORF) encoding the protein of interest, flanked by 5’ and 3’ untranslated regions (UTR’s) (Fig. 1). The poly(A) tail at the 3’-end and Cap at the 5’-end are critical for the mRNA stability. Further, the cap is essential for translation efficiency. Also the poly(A) tail and the 3’ and 5’ UTRs are important to increase translation efficiency and mRNA half-life [28–32]. The poly(A) tail length can influence translation efficiency and affects the mRNA decay. In practice, a tail length of approximately 100 nucleotides (nt) is widely used, but the optimal length may vary depending on mRNA construct and application [28, 33–35].

The manufacturing begins with the design and production of a circular plasmid DNA template having a promotor for the used bacteriophage polymerase, e.g. T7 RNA polymerase, followed by the encoded sequence. The poly(A) tail can either be encoded into the plasmid sequence, added to the template by PCR or added to the mRNA enzymatically post IVT [3, 36].

**Fig. 2:**
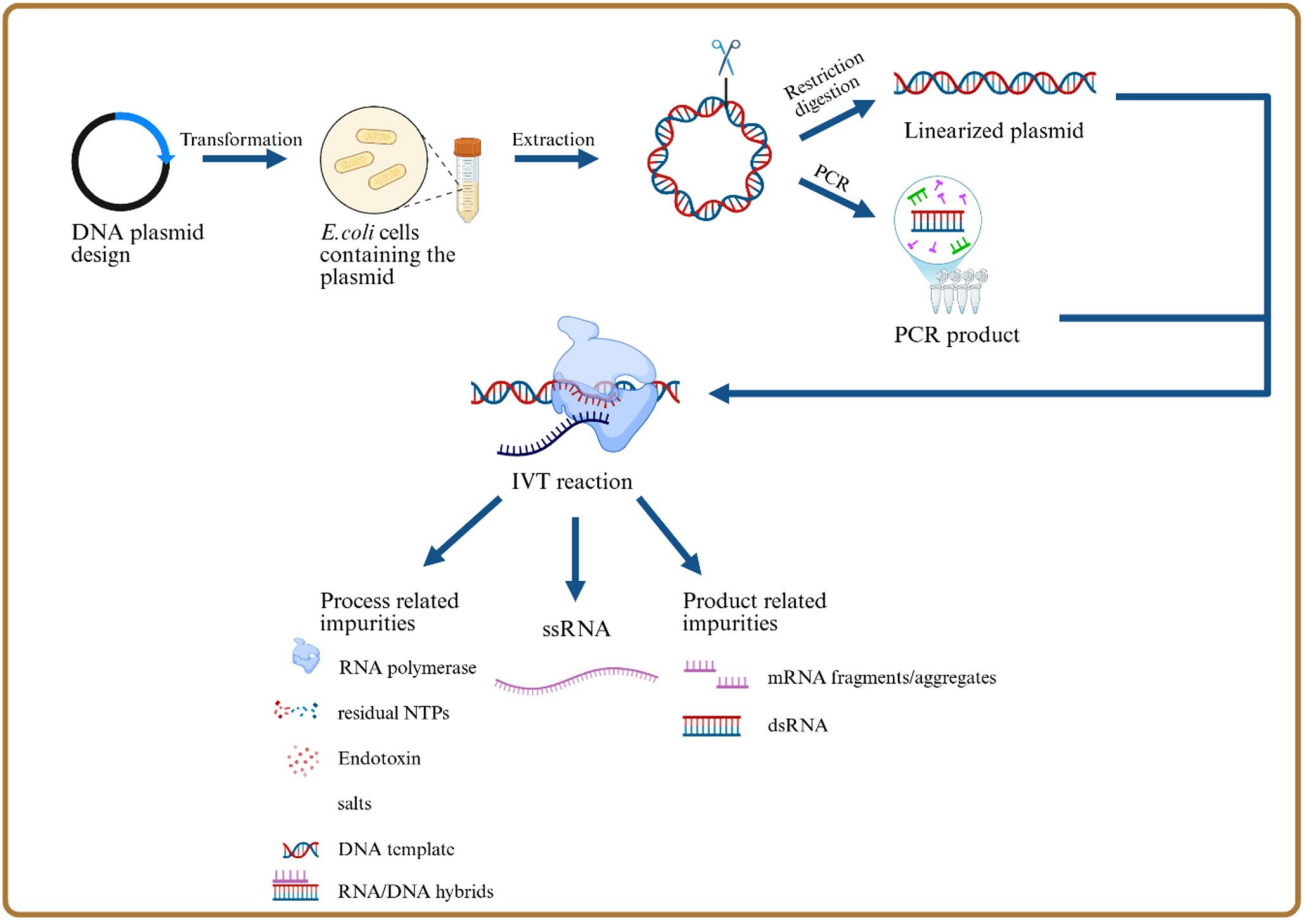
Schematic illustration of the mRNA manufacturing process. Starting with the plasmid DNA design which will be transformed into competent *E. coli* cells. After plasmid extraction either a restriction digestion is performed to linearize the plasmid, or a PCR. Both templates are suitable to perform the IVT reaction. The outcomes of the IVT are the desired ssRNA and process related impurities like residual NTPs, DNA template, RNA-DNA hybrids, RNA polymerase, endotoxin and salts as well as product related impurities like fragments, aggregates, and dsRNA. Created in BioRender. Nowak, C. (2025) https://BioRender.com/ls3j7ka

The DNA template used for mRNA production is first produced in *E. coli*, then extracted and prepared either by linearizing the plasmid with restriction enzymes or by amplifying it through polymerase chain reaction (PCR). Both forms, linearized plasmids and PCR products, are suitable for IVT. It’s important to linearize the DNA just after the mRNA-coding region because the natural T7 terminator is weak. If the plasmid remains circular, the T7 RNA polymerase may keep transcribing into unwanted regions, resulting in longer, non-functional mRNA. By linearizing the template or using PCR products, transcription stops precisely at the intended site, producing what’s known as a “run-off” transcript. This ensures that the mRNA is the correct length. While the native T7 terminator only stops transcription about 62% or even less of the time, its efficiency can be increased to up to 98% with certain modifications [37, 38]. A schematic illustration of the mRNA manufacturing process is shown in Fig. 2.

The RNA polymerases commonly used to produce mRNA are T3, T7 and SP6 [39]. The most frequently used RNA polymerase is bacterio phage T7 RNA polymerase which is a 99 kDa enzyme [40]. T7 RNA polymerase is favoured in mRNA manufacturing due to its high transcriptional fidelity and high yield [10, 11].

IVT can be divided into three stages, -initiation, elongation and termination. During initiation, T7 RNA polymerase forms an initiation complex by binding to the promotor. This complex is known to be unstable and frequently produces short, abortive mRNA fragments of 2–10 nucleotides in length [11, 41–43]. Once the transcription exceeds approximately 10 nucleotides, the T7 RNA polymerase releases the promotor region and forms a stable elongation complex [40, 42–45]. Upon reaching the end of the linearized template the polymerase falls off the template, resulting in a process known as “run-off transcription” [40, 46–50]. At this stage, full-length RNA is produced alongside RNA-related by-products, including abortive fragments from incomplete transcription, dsRNA and aggregates [10, 11, 40].

Following transcription, the mRNA must be purified to the desired purity, removing free nucleotides. Chromatographic purification methods for mRNA have been reviewed in detail [51].

## dsRNA

### Formation of dsRNA

**Fig. 3:**
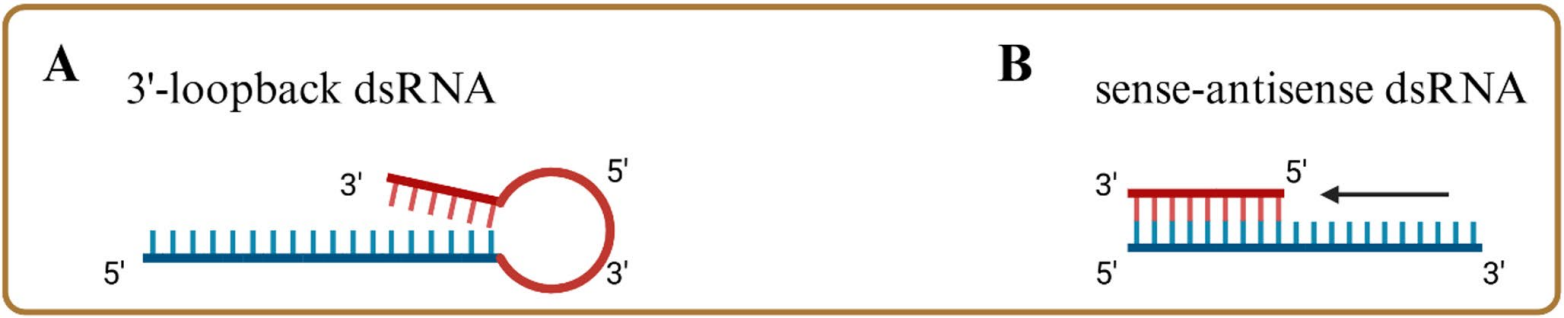
The two proposed types of dsRNA impurities formed during IVT. (A) shows the 3’-loopback dsRNA, the target sequence is coloured in blue, the annealed sequence in red. (B) shows the sense-antisense dsRNA which is formed by annealing of complementary sequences. Created in BioRender. Nowak, C. (2025) https://BioRender.com/s6el0kv

Studies proposed two main types of dsRNA molecules (Fig. 3) [12, 14]. The first type is 3’-loopback dsRNA, where a single RNA molecule folds back and hybridizes with itself, forming extended regions of intramolecular base pairing (Fig. 3A). The second type is sense–antisense dsRNA, in which two complementary RNA molecules bind to each other to form the double-stranded structure (Fig. 3B).

The exact mechanism of dsRNA formation during IVT is not yet fully understood. However, one study hypothesized three possible reaction pathways during IVT. The first, termed the “on-pathway”, occurs early in the IVT process when T7 RNA polymerase binds to the promoter and initiates transcription, producing canonical run-off transcripts. The second, the “off-pathway”, involves previously synthesized RNA hybridizing with itself or acting as a template for unintended RNA extension, potentially leading to the formation of 3′-loopback dsRNA. The third, named as “pseudo-on pathway”, is sequence-dependent and may occur when specific motifs at the 3′ end of the template DNA facilitate the synthesis of antisense transcripts, which can then hybridize with the sense strand to form sense–antisense dsRNA [12].

The exact mechanism on why 3’-extended mRNA is being formed is unknown, but a likely contributing factor is a loose 3’-end that lacks stable secondary structure, allowing the polymerase to use it as a template in place of the DNA for further extension [52]. This self-extension can result in intramolecular (cis) duplexes when the RNA folds back on itself, or intermolecular (trans) duplexes when it anneals to another RNA molecule with complementary sequences [12, 14, 52–56].

This 3’-loopback dsRNA has been identified as the predominant byproduct produced in IVT reactions [12, 53]. The formation of 3’-loopback dsRNA has been shown to be sequence-dependent with homopolymeric runs prone near the 3’terminus being particularly prone to self-priming and dsRNA formation [52, 53].

The second type of unwanted dsRNA formation arises from transcription initiation on the non-template strand of DNA, producing antisense RNA that pairs with the sense RNA transcript [12, 14]. This promoter-independent antisense transcription has been observed across a wide range of DNA sequences and appears to depend on specific motifs at the 3’-end of the DNA template [14]. These sequences can cause T7 RNA polymerase to initiate transcription on the non-template strand, generating antisense RNA and leading to sense–antisense dsRNA formation.

Importantly, the inclusion of a poly(T) sequence in the DNA template has been shown to reduce the formation of antisense dsRNA by-products, though it does not affect 3’-loopback dsRNA formation [12]. The precise mechanism remains under investigation. Wu et al., propose that certain 3’-terminal sequences promote strand-switching by T7 RNA polymerase, triggering antisense transcription. In the absence of these antisense-promoting motifs, 3′ self-extension and loopback-mediated dsRNA formation tend to predominate [12].

### Impact of dsRNA

Due to their immunological impact the translation efficiency is low if mRNA therapeutics with dsRNA contamination are used. The innate immune system detects viral RNAs, such as dsRNAs, through pattern recognition receptors (PRRs). The signature of viral RNAs is the RNA duplex structure which initiate antiviral immune response through cytosolic sensors, retinoic acid-inducible gene I (RIG-I) and melanoma differentiation-associated 5 (MDA5), the two major sensors [14, 22, 57]. If viral dsRNA is recognized by RIG-I or MDA5 it activates the antiviral signalling pathways followed by the production of interferons type I and III (IFNs). Both receptors are similar in their domain structures and amino acid sequences but have different substrate preferences and mode of filament formation. MDA5 binds long dsRNA in a sequence dependant manner by interacting with the backbone of the RNA duplex. In contrast, RIG-1 recognizes the 5′-triphosphate (5′ppp) or 5′-diphosphate (5′pp) moieties at the ends of short dsRNA or blunt-ended duplexes [57].

Because T7 RNA polymerase transcripts naturally carry 5′ppp groups, they are inherently immunogenic and capable of activating RIG-I [14]. But removal of 5’ppp does not completely suppress the immunogenicity which can be explained by MDA5 activation which is independent of 5′ppp and responds primarily to the presence of dsRNA itself [14, 58].

Due to the triggering of the innate immune system dsRNA contaminations in mRNA therapeutics can lead to severe side-effects in the patients’ body. Also, in vitro and in vivo studies can be affected, as triggering of the immune response shuts down the protein synthesis pathway and hence leads to lower translation efficiency [3, 14].

### Mitigation of dsRNA

Various studies investigated how the formation of dsRNA can be reduced during IVT reaction (Table 1), which are described in the following.

#### DsRNA mitigation by introducing modified nucleotides

In one study of Mu & Hur, they replaced original nucleotides with chemically modified nucleotides during IVT and observed that antisense-mediated dsRNA byproducts could be reduced when pseudouridine, 1-methylpseudouridine and m5C were incorporated [57]. These results are consistent with those of other studies [21, 57]. Therefore, modified nucleotides can in part reduce the immunogenicity of in vitro-transcribed RNA by inhibiting the production of dsRNA byproducts.

The mechanism how modified nucleotides suppress dsRNA formation is still unclear [57].

Several modified nucleotides, such as pseudouridine, N1-methylpseudouridine, 6-methyladenosine (m6A) or 5-methylcytosine (m5C) could supress RIG-1 activity and increase the translation efficiency. The MDA5 activity was unaffected by using modified nucleotides, which could be explained by its non-interacting with bases, but its binding to the dsRNA backbone [57].

#### DsRNA mitigation by adjusting IVT reaction components

The results regarding the positive effects of modified nucleotides on lowering immunogenicity of T7 transcripts lead to the suggestion that the transcription reaction components might influence dsRNA byproduct formation. Another study of Mu et al. was performed using different concentrations of selected reaction components, including DNA template, T7 RNA polymerase, NTP, NaCl and MgCl_2_ [14].

They found that dsRNA formation is dependent on the T7 RNA polymerase concentration, also NTP and NaCl concentration had a small impact. Decreasing the concentration of MgCl_2_ from 30 mM to 5 mM reduced the dsRNA formation significantly, while the total yield of RNA was not significantly affected [14]. These results suggested that MgCl_2_ can play a role in dsRNA synthesis regulation.

#### DsRNA mitigation by high ionic strength

Another study tried to prevent the 3’ self-extension of the RNA [59]. For this, the IVT was performed under high salt concentrations to reduce electrostatic protein-nucleic acid interactions, between RNA and the polymerase, thereby reducing undesired non-template primed extension. However, the increased salt concentration completely inhibited the enzymatic activity of T7 RNA polymerase. To overcome this, both the polymerase and the DNA template were brought into close spatial proximity by independently immobilizing them, via flexible linkers, onto specially engineered magnetic beads coated with Strep-TactinXT.

With this method it was possible to bring the T7 RNA polymerase in close proximity to its promoter and thus to restore promoter binding at high ionic strength.

In this tethered reaction high salt inhibited only the unwanted cis-primed extension activity of T7 RNA polymerase resulting in a significantly decrease in the yield of longer, double-stranded impurities. This resulted in higher yields of the desired RNA with greater purity. An optimum of 0.3 M NaCl was observed for the tethered system [59].

It is noteworthy that this tethered system has been only used for short RNAs (24 and 34 nucleotides). According to preliminary results, the system used in this study was not able to bind > 120 base DNA templates efficiently [59].

#### DsRNA mitigation by purifying DNA template prior IVT

The quality of the linearized DNA template used in IVT also plays a crucial role in dsRNA formation as Martinez et al. has shown [60]. They purified the linearized plasmid by C4 RP-HPLC.

The purified templates were then used for IVT reactions, and a significant reduction of dsRNA was observed.

It is suggested that the purification of the linearized template removes impurities of DNA that can serve as a template to produce RNA fragments complementary to the original transcript which can then anneal to the properly transcribed RNA and thus generate dsRNA byproducts [60].

These findings highlight the significant impact of process-related impurities on the generation of dsRNA and potentially other RNA-related byproducts such as RNA fragments and dsRNA. While the use of purified, linearized DNA templates appears to be an effective strategy to reduce dsRNA levels, it does not completely eliminate their formation.

The dsRNA formation pathways described above suggest promoter-independent transcription, which aligns with observations reported by Martinez et al. Their study indicates that DNA template impurities can also act as unintended templates for T7 RNA polymerase in a promoter-independent manner.

#### DsRNA mitigation with chaotropic reagents

The study by Piao et al. (2022) investigated the role of RNA thermodynamics in dsRNA formation during in vitro transcription (IVT). They proposed that both mechanisms of dsRNA formation, 3’-loopback and sense–antisense pairing, originate from undesired inter- or intramolecular base pairing. Based on this, they hypothesized that reducing such base-pairing interactions could lower dsRNA levels during IVT.

To test this, they introduced mild chaotropic agents, such as formamide and urea, into the IVT reaction to disrupt RNA duplex formation. The mRNA synthesized contained N1-methylpseudouridine modifications, commonly used to reduce immunogenicity. Both formamide and urea significantly reduced dsRNA formation, approaching levels seen with HPLC-purified mRNA, particularly at concentrations of 1.6 M formamide and 0.8 M urea. Urea was especially effective, reducing dsRNA content by approximately 60–70%, as measured by J2 monoclonal antibody (mAb) dot blot analysis.

Importantly, T7 RNA polymerase activity was only mildly affected, as overall RNA yields remained high. Additionally, the mRNA produced under these conditions exhibited enhanced translation efficiency, as expected [61].

#### DsRNA mitigation with engineered T7 RNA polymerase

Another approach was to engineer T7 RNA polymerase to produce mRNA free of immunostimulatory by-products [40].

They focused on the C-terminal Phe-Ala-Phe-Ala^883^, a conserved motif which is situated within the carboxyl-terminal domain. It has been shown that the residues, when mutated, influence the initiation and elongation rates [62–64]. Through computational modelling, structural and mechanical laboratory approaches they developed a double mutant - G47A and 884G, which showed a decrease in short and loopback dsRNA when compared with wild-type T7 RNA polymerase, but minimal difference in amounts and composition of the abortive transcripts [40].

#### DsRNA mitigation by introducing thermostable T7 RNA polymerase

In vitro transcription (IVT) at elevated temperatures, above 48 °C, using a thermostable T7 RNA polymerase has been shown to reduce the formation of 3’-extended byproducts. However, high-temperature IVT had little to no effect on the generation of antisense RNA byproducts. This suggests that elevated temperatures primarily prevent RNA from rebinding to itself, thereby reducing intramolecular base pairing, but do not inhibit promoter-independent antisense transcription [12].

From a manufacturing perspective, some mitigation strategies to decrease dsRNA formation during IVT reaction are quite feasible. Decreasing MgCl_2_ can be easily conducted at low-cost. It would be worth to investigate how other IVT components influence the dsRNA formation, since changing only the IVT reagents needed for mRNA synthesis would be the fastest and the most cost-effective mitigation method. Replacing nucleotides with modified ones also resulted in reduced dsRNA levels and is used in the production of the COVID-19 vaccines from Moderna and BioNtech/Pfizer [65].

Other mitigation methods like immobilizing T7 RNA polymerase and DNA template in close proximity, while increasing NaCl concentration to suppress non-specific interactions, is an interesting approach but needs further investigation for manufacturability. Although promising, this approach requires further evaluation to determine its feasibility for large-scale manufacturing. Since this approach may not bind DNA templates > 120 base pairs, it is not suitable to be used for longer RNAs such as mRNAs used for therapeutic applications at this stage. Cavac et al. assumed that this method could be used to synthesize long mRNAs if the DNA is immobilized properly [59].

Engineering a novel T7 RNA polymerase, as demonstrated by Dousis et al., is a time- and resource-intensive approach that requires specialized equipment that may not be readily accessible in most manufacturing environments. As a result, for large-scale or routine production settings, it is more practical to optimize the IVT reaction using currently available commercial T7 RNA polymerases rather than developing custom-engineered enzyme [40].

**Table 1:** Summary of scalability and limitations of different dsRNA mitigation strategies

Mitigation strategy	Industrial scalability	Limitations
Improve IVT reaction (e.g. using different concentrations of reaction components such as Mg^2+^) (14)	High	Time - the ideal reaction conditions have to be investigated for each IVT system, and might be influenced by the sequence
Using modified nucleotides (57)	High	Costly - modified nucleotides are more expensive than unmodified ones, but they are highly effective
Using chaotropic reagents (61)	Middle	Unknown effects on other mRNA impurities
High-temperature IVT (12)	Low	High risk of degradation of desired ssRNA
Immobilization of template and enzyme at elevated NaCl concentration (59)	Low	Costly and not suitable for long therapeutic mRNAs
Purification of plasmid DNA template (60)	High	Time - as an additional step prior IVT has to be implemented
Engineering new T7 RNA polymerase (40)	Low	Reliant on access to engineered T7 RNA polymerase

### Analytics of DsRNA

The content of dsRNA impurities is commonly measured with immuno-Dot Blot assays or ELISA [66, 67]. Both techniques rely on monoclonal antibodies that specifically recognize dsRNA structures. The dot blot assay, while conceptually similar to a Western blot for proteins, is adapted for nucleic acids and is frequently used in academic research settings [21].

Native PAGE using either Sybr Gold or acridine orange can be used to visualize single- or double stranded RNA or DNA in native polyacrylamide gels.

It binds the charged backbone of nucleic acids and increases the fluorescence signal by more than 1000-fold [68]. Acridine orange (AO) is a dye that binds RNA regardless of its secondary structure but fluoresces differently depending on its secondary structure (14). With a gel scanner, the fluorescent gel images can be obtained, and AO emits green fluorescent when bound to ssRNA and red fluorescence when bound to dsRNA.

This method can be easily used to visualize and distinguish between ssRNA and dsRNA after IVT reaction, but it gives no information about the other by-products. Native PAGE is time-consuming, semi-quantitative, and low-throughput, making them less suitable for process development or GMP manufacturing workflows. More efficient, high-throughput, and quantitative analytical methods are needed to support scalable mRNA production.

**Fig. 4:**
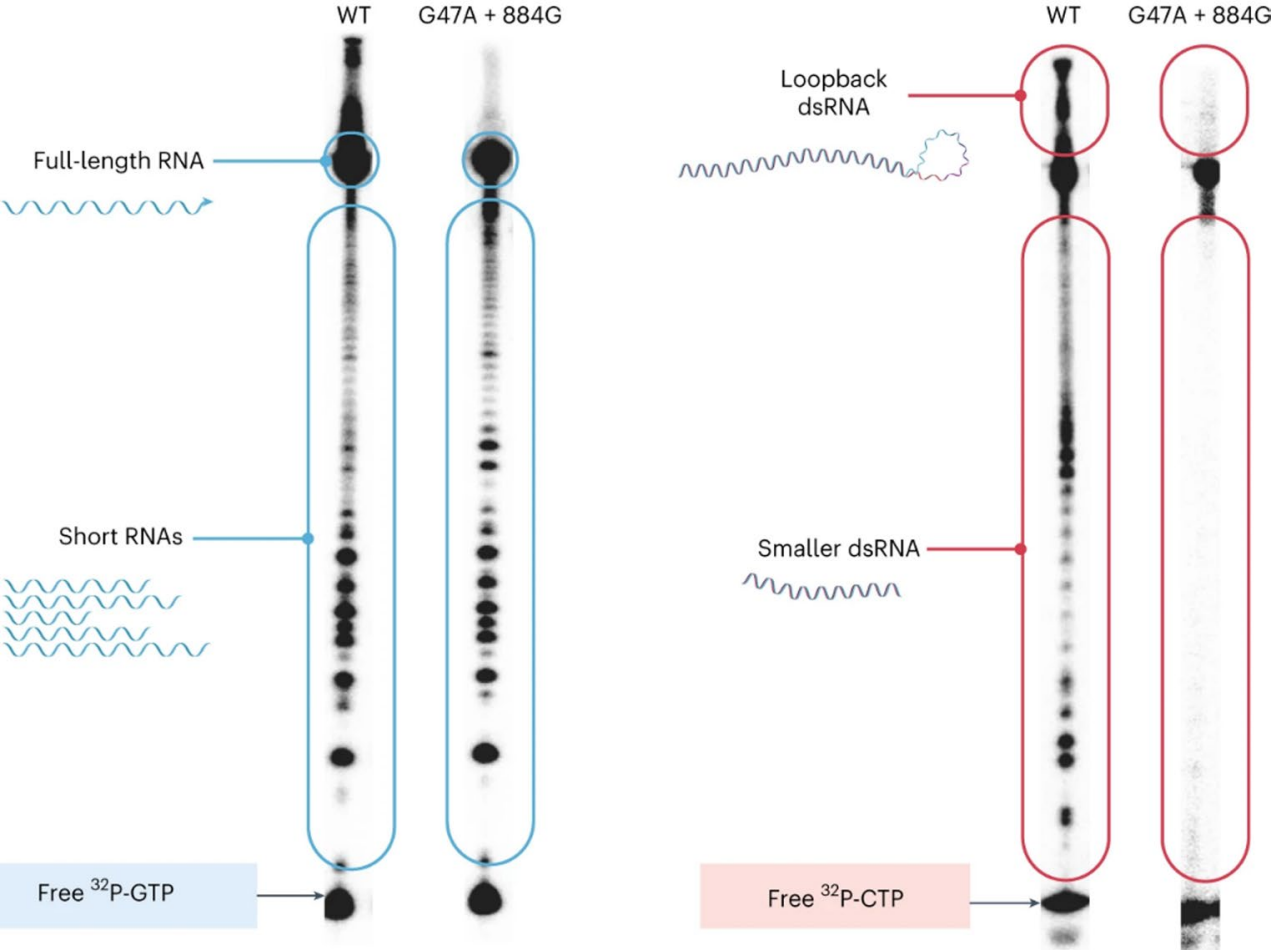
Analysis of mRNA impurities, produced from the wildtype T7 RNA polymerase and a new engineered one (G47A + 884G) via gel electrophoresis using radioactive labelled nucleotides. Reprinted and modified with permission from [40].

A very sensitive method to detect not only dsRNA but also other impurities produced by T7 RNA polymerase, such as RNA fragments, is the radioactive gel electrophoresis in which ^32^P-labeld NTPs. In this approach, a DNA template is designed with G-rich 5′ region followed by C-rich sequences later in the transcript. With α-^32^P-GTP abortive species and full-length RNAs can be visualized, whereas IVT reactions that incorporates α-^32^P-CTP label short and long dsRNAs (Fig. 4) [40]. The sample can then be analysed by gel electrophoresis.

A major disadvantage of this analytical method is the cost: ^32^P-labeld NTPs are very expensive, and additional security measures are required, such as a Geiger counter and a separated workspace. It also requires a defined template sequence. These factors make the method less suitable for many laboratories.

**Fig. 5:**
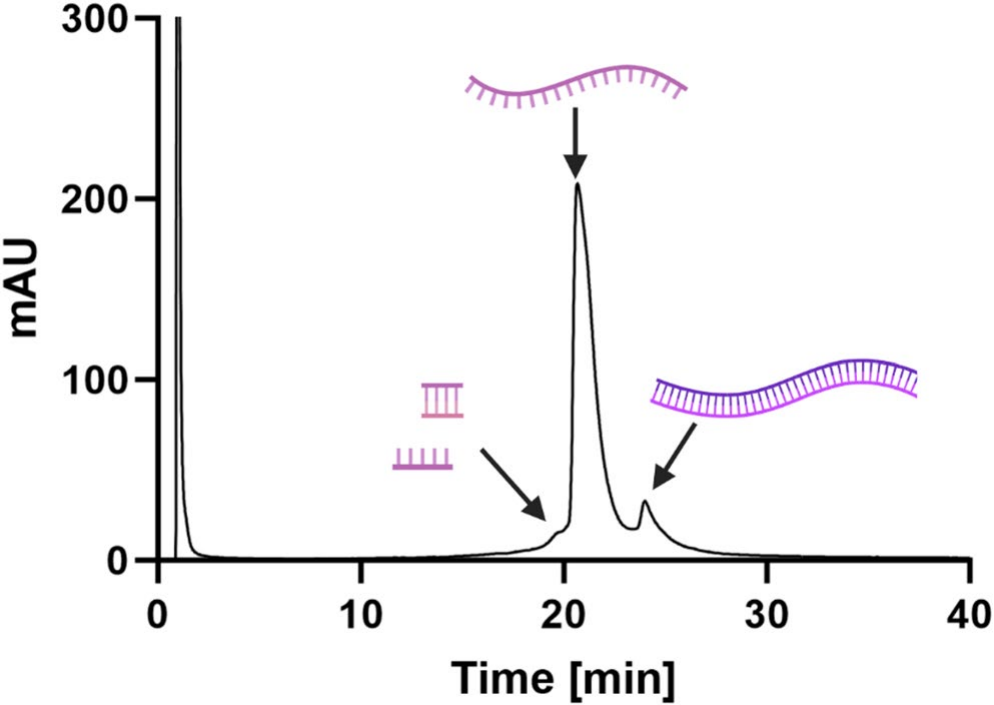
IP-RP-HPLC analysis of ssRNA and impurities. Shown is a chromatogram of a crude IVT sample analysed at 260 nm and increasing acetonitrile gradient. Created in BioRender. Nowak, C. (2026) https://BioRender.com/aq5n4h7

In addition to molecular and immunobiological methods, chromatographic techniques have become well-established tools for the analysis of mRNA impurities, offering higher precision resolution, and quantifiability, —qualities particularly advantageous in a manufacturing and quality control context. One widely used technique is ion-pair reversed-phase high-performance liquid chromatography (IP-RP-HPLC), wherein the negatively charged sugar-phosphate backbone of mRNA interacts with quaternary ammonium ion-pairing agents present in the mobile phase [2, 69, 70]. This interaction increases the hydrophobicity of the RNA molecules, allowing them to bind to the nonpolar stationary phase of the reversed-phase column [71]. Analysis is typically performed under denaturing (75 °C) or partially denaturing (50 °C) conditions to resolve structural variants and by-products with on-line UV detection at 260 nm to monitor RNA species [72]. mRNA forms intramolecular loops and hairpins via base pairing which would lead to poor resolution and broad peaks. Hence, performing this analytical technique under 50–75 °C degrees is necessary to denature these structures into a more linear form which results in sharp peaks as shown in Fig. 5.

This method offers several advantages for mRNA impurity profiling. Process-related impurities, such as residual nucleoside triphosphates (NTPs), buffer components, and capping reagents, are not retained on the reversed-phase column and are thus readily separated from the mRNA product [73].Oligonucleotides are typically resolved based on size and secondary structure in IP-RP-HPLC, with longer sequences exhibiting increased retention times. As a result, product-related impurities such as short abortive transcripts, RNA fragments, short dsRNA, and full-length dsRNA can be effectively distinguished from the target single-stranded mRNA (ssRNA) [74, 75]. Notably, dsRNA displays greater hydrophobicity than ssRNA, leading to longer retention times and allowing its identification as a distinct peak in the chromatogram (Fig. 5) This has been shown by several studies [73, 75].

In a typical IP RP-HPLC chromatogram, the initial peak corresponds to process-related impurities, such as salts, unincorporated nucleotides, and capping agents, which are not retained on the column. Subsequent smaller peaks represent short RNA fragments, abortive transcripts and short double-stranded RNA (dsRNA), which elute earlier due to their lower molecular weight, structure and reduced hydrophobicity. The main peak corresponds to the desired single-stranded mRNA (ssRNA), followed by a later-eluting peak representing dsRNA, which exhibits increased hydrophobicity and retention time.

Despite its utility, IP-RP-HPLC has certain limitations, primarily associated with the resolution capacity of the column. Short abortive transcripts often produce overlapping or poorly resolved peaks. Moreover, longer aggregates or truncated dsRNA species with similar hydrodynamic properties, such as similar structure, to ssRNA may co-elute, making them difficult to distinguish. While the commonly used ion-pairing agent, triethylammonium acetate (TEAA), adds to the operational cost, IP-RP-HPLC remains one of the most rapid and reliable methods for detecting dsRNA in both manufacturing and quality control environments.

Additional critical quality attributes include mRNA sequence fidelity, structural identity, and poly(A) tail integrity. Emerging sequencing methods, such as the VAX-seq a nanopore-based, long-read cDNA sequencing protocol, enable comprehensive analysis of these features [76]. This approach detects mismatches introduced during IVT, characterizes poly(A) tail length and composition, and identifies degradation products and byproducts such as dsRNA. VAX-seq employs a 3’ reverse adaptor for accurate poly(A) tail measurement and has shown greater reliability than direct RNA sequencing. Its low mRNA input requirement makes it suitable for routine quality control across manufacturing stages, including DNA template validation.

## mRNA fragments

### mRNA fragment formation

The production of mRNA fragments can be caused by the catalytic cycle of T7 RNA polymerase. The first step of IVT is initiation. The T7 RNA polymerase binds to the promotor forming the initiation complex (IC), which is relatively unstable. In this stage, as a part of early initiation, short abortive mRNAs are produced [40, 77].

Several studies in the past could reveal that the production process of abortive transcripts during the initiation stage, is a fundamental part of transcription. This process is named abortive cycle and has also been described for other RNA polymerases [43].

After the incorporation of about 10 nucleotides the ternary enzyme-DNA-RNA complex transits into the processive and more stable elongation complex (EC) [40]. This transition marks the end of the abortive initiation phase and the onset of productive RNA synthesis.

Martin et al. concluded that the abortive cycle is dependent on temperature and ionic strength [43]. The abortive cycling increased with increasing temperature and ionic strength but the amount of abortive transcripts varied depending on the initial sequence of the message (around 8–10 bases).

During the initial phase of transcription, two competing events can occur at each nucleotide position: the ternary enzyme–DNA–RNA complex may either dissociate, releasing a short abortive transcript, or successfully incorporate the next nucleotide. Martin et al. proposed a kinetic competition between nucleotide incorporation and dissociation of the ternary complex. Although dissociation is possible during initiation, elongation is generally kinetically favored, even in the earliest steps, and becomes strongly dominant once the complex transitions to a fully processive elongation state [43].

Approximately 90% of the transcripts that have escaped the abortive cycle successfully produce full-length transcripts of a 10,000-nucleotide template [43]. The results presented in this study suggest, that immediately after initiation the enzyme-DNA-RNA ternary complex possesses a higher probability of dissociation that after transition to a fully processive complex. Martin et al. provided three possible reasons for the transition from an unstable initiated complex to a highly processive ternary complex. The first hypothesis is that the formation of a DNA-RNA duplex stabilizes the ternary complex with increasing RNA length until the origin of the DNA-RNA duplex dissociates. Another reason could be a conformational change of the protein to an altered form with higher processivity. Other studies revealed this conformational change after synthesis of between 6 and 15 bases [43, 78]. Lastly, they proposed that nonspecific interactions between specific regions of the protein and the growing RNA transcript could stabilize the ternary complex. Assuming that the RNA-binding domain of the protein is spatially separate from its catalytic site, stabilization would not occur until the RNA chain reaches a certain length to allow stabilization interaction [43].

**Fig. 6:**
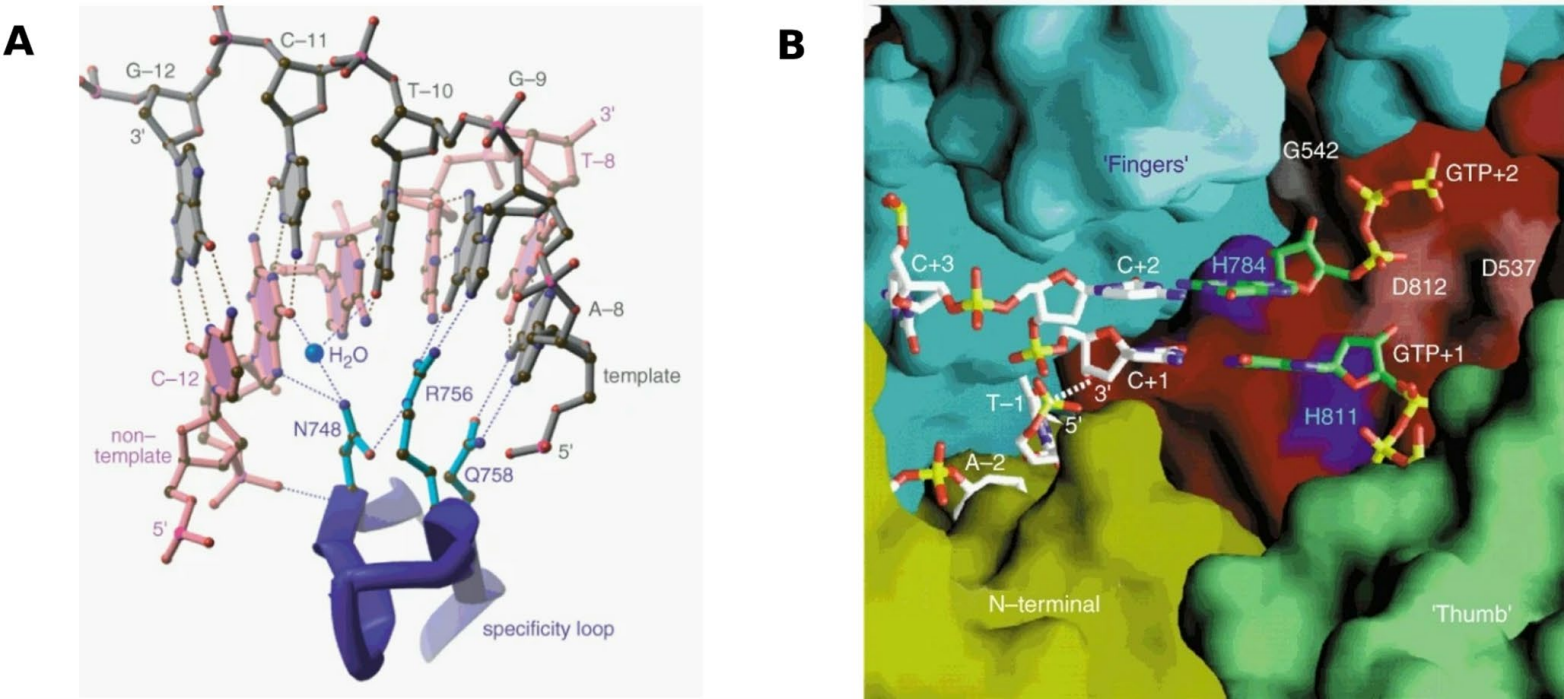
(**A**) Crystallized structure of interactions between the specificity loop of the T7 RNA polymerase and the T7-promoter DNA. The template strand is coloured in grey, the non-template strand in pink. The hydrogen-bonding interactions between the side-chains of this loop (turquois) and the bases of the promoter are shown. Another part of the T7 RNA polymerase-DNA interface is a bound water molecule. Reprinted and modified with permission from [11]. (**B**) Modelled initiation complex with the structural components of T7 RNA polymerase, the N-terminal, Thumb and Fingers. This represents the active site cleft of T7 RNA polymerase. Coloured in cyan are the histidine residues. Observed are also nucleotides of the template strand − 2 and − 1, priming (+ 1) and incoming (+ 2) GTPs and the complementary template strand + 1 to + 3. Reprinted and modified with permission from [11]

On a structural basis, the T7 RNA polymerase uses a specificity loop, an antiparallel β-hairpin motif with four side-chains on one side which is part of the N-terminal domain of the enzyme, to interact with the base pairs of the T7 promoter. This forms the basis for differentiation of T7 promoter and other DNA sequences [11]. Figure 6A shows the interactions between the T7 promoter and the specificity loop.

Melting the promoter duplex in order to initiate transcription is eased by the N-terminal of the T7 RNA polymerase. In this mechanism, which is called the transcription bubble [45], another complex called the intercalating hairpin is involved [11]. Due to interactions between residues of the specificity loop and the template strand in an open conformation the promoter is stabilized. It is important to note that the hydrogen-bonding interactions between the loop and the promoter bases, shown in Fig. 6A, are sequence-specific [11]. In the same study, Cheetham et al. could also create a model of the initiation complex based on the homology of T7 RNA polymerase and DNA polymerases which is shown in Fig. 6B.

This model suggested that the T7 RNA polymerase positions the DNA template strand exactly in its active site and therefore provides complementary base-pairing sites for the priming and incoming NTPs at the + 1 and + 2 positions.

Based on these findings, Cheetham et al. proposed the abortive cycling occurs due to accumulation of the template strand in the active site while the promoter duplex is still bound to the enzyme [11].

Several studies have demonstrated that a conformational change of the polymerase plays a crucial role in the transcriptional processivity. It is hypothesized that with increasing RNA length (after 3–7 nt) the RNA-DNA hybrid is ‘pushing’ on the N-terminal driving it to rotate around 40° which causes a structural change in the enzyme leading to weaker promoter contacts. This triggers the promoter release and forms the RNA exit channel. However, the enzyme must push back on the RNA-DNA hybrid which leads to destabilization of the latter and results in the abortive loss of the RNA [42, 50, 79–81].

After 9 to 12 nt are synthesized a drastic 220° rotation of the enzyme occurs which causes a release of the upstream promoter interactions resulting in the transition of T7 RNA polymerase into the stable elongation complex. The abortive transcripts produced during the initiation phase can have a length of 2 to 13 nt [50, 79, 80, 82–87].

**Fig. 7:**
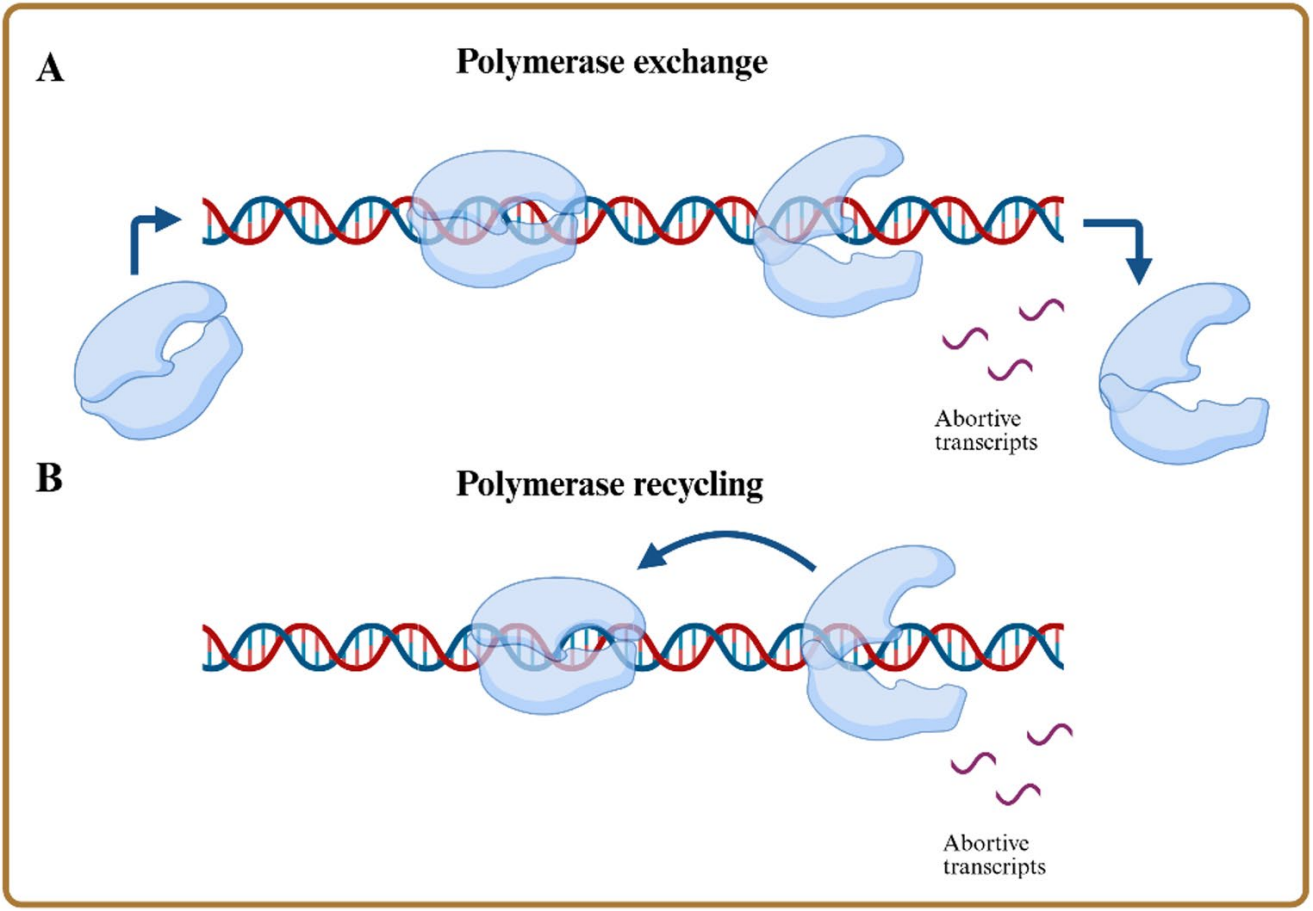
Schematic illustration of the two possible pathways for the formation of abortive transcripts. (**A**) ‘polymerase exchange’: T7 RNA polymerase releases abortive transcripts, dissociates and a new T7 RNA polymerase binds onto the DNA template. (**B**) ‘polymerase recycling’: after releasing abortive transcripts the same T7 RNA polymerase rebinds onto the template and re-starts RNA synthesis. Created in BioRender. Nowak, C. (2025) https://BioRender.com/ydradh2

The transcription initiation process was studied at near base-pair resolution [88]. They concluded two possible pathways to produce abortive transcripts (Fig. 7). First, the ‘polymerase recycling’ in which the T7 RNA polymerase re-starts RNA synthesis after releasing abortive transcripts while remaining bound to the template strand. Second, the ‘polymerase exchange’ where the T7 RNA polymerase with or after RNA release dissociates from the DNA and synthesis of a new RNA starts only after a new T7 RNA polymerase binds on the DNA [88].

Another important investigation of this study was that both pathways can occur on the same DNA molecule and the partitioning between both is sequence dependent. At high concentrations of GTP a preference of the polymerase recycling pathway was observed, probably due to kinetic interactions between synthesis and T7 RNA polymerase dissociation [88]. This leads to the suggestion that nucleotide availability, especially GTP, can modulate the choice of abortive transcript production pathway.

based on the description of Koh et al. [88].

It is noteworthy that the presence of diphosphate (PPi) plays an important role during the initiation process. One study investigated the impact of diphosphate on the formation of abortive transcripts and found out that with increasing diphosphate the amount of abortive transcripts also increases [89].

Besides the formation during IVT, also hydrolysis of the mRNA or contamination with RNase can lead to the formation of fragmented mRNAs [24].

Hydrolysis likely occurs due to broken phosphodiester bonds that form the mRNA backbone. This is caused by a nucleophilic attack by a 2’OH group on the phosphate ester bond [90]. Previous studies observed that the secondary structure of the mRNA as well as the base sequence influence the rate of hydrolysis [91, 92]. Some cations like Mg^2+^ can also increase the activity of certain RNases which leads to hydrolysis of mRNA [93, 94].

These short mRNAs can interact with T7 RNA polymerase via promotor independent RNA-templated transcription, producing short complementary transcripts and forming dsRNAs.

Metal ions can have a crucial influence on the RNA secondary structure, its folding and stability [95, 96]. Contaminations with metal ions can occur due to storage bags, containers, etc [85]. Several studies revealed the possible impact of different metal ions on mRNA and some can cause hydrolysis of RNA due to the binding of sequence-specific metal ions such as Pb^2+^ (lead(II) ion) [85, 97].

### The impact of mRNA fragmentation

Short mRNA fragments decrease mRNA purity and yield. Due to the incompletion of the IVT reaction the mRNA that is produced at this stage is truncated, so they are unable to translate the complete ORF resulting in reduced translation efficiency [24]. The more mRNA fragments are produced during manufacturing the lower is the potency of the therapeutics and therefore the therapeutic success for the patient. Besides translation efficiency mRNA fragments have also an impact on immunogenic responses since also short dsRNAs are formed [40] and therefore can trigger the cytosolic sensors of the immune system in the same way as long dsRNAs [14, 22, 57].

### Mitigation of mRNA fragmentation

Because of the impacts of mRNA fragments described above it is necessary to remove those fragments or prevent their formation during IVT [2, 98].

Studies suggested that lowering the initiation complex to elongation complex transition barrier can reduce the production of short RNAs. As described in 3.3, one research group recently showed that T7 RNA polymerase can be engineered to produce mRNA free of immunostimulatory by-products and short mRNA fragments [40].

One such engineered variant, P226L, proved to drastically reduced mRNA fragments with a length of 4–7 nt [79, 99, 100]. However, longer fragments (9–11 nt) were still presented, suggesting that the transition from IC to EC was not fully stabilized with this mutant alone.

A hypothesis was that weakening the T7 RNA polymerases’ interactions with the promotor would facilitate the promotor release, leading to a higher stability of the RNA-DNA hybrid and therefore reduced the formation of fragmented mRNA.

A combination of P226L modified T7 RNA polymerase and a modified promoter that weakens upstream promoter interactions reduced all species of abortive transcripts. This modified promoter had a cysteine instead of an adenosine and is called ϕ9(A-15 C). It was observed that this modified promoter was able to reduce longer abortive byproducts by facilitating the P266L mutant to undergo the transition from IC to EC at 9 nt [85, 100].

A study also investigated the use of truncated promoters to mitigate formation of abortive transcripts, but IVT reactions with truncated promoters led to similar levels of abortive byproducts [101].

Various promotor topologies were tested for reduced mRNA fragment production. These included, -insertion of six non-complementary nucleotides in the non-template strand (between pos. −5 and − 4) and non-complementary non-template strands on position − 4 to + 5. Additionally, nicks in the template strand between position − 5 and − 4 have been tested. Only the nicked DNA template strand on position − 5 resulted in significantly reduced mRNA fragments but also led to a reduced overall mRNA yield [102]. Thus, using truncated or nicked promotors to reduce fragment formation are not suitable strategies for mRNA manufacturing.

The presence of pyrophosphate (PPi) can increase the level of abortive transcripts. Therefore, the inclusion of pyrophosphatase into the IVT reaction can reduce the formation of these byproducts. Several studies showed that pyrophosphatase increased the total mRNA yield which made it an even more attractive mitigation strategy [103, 104].

Another approach that can help improving mRNA quality is the development of mRNA with enhanced structural properties that transit more rapidly into the elongation complex or exhibit reduced loopback folding [105].

This can be aided by novel artificial intelligence and machine learning (AI/ML) algorithms, like PETfold or FARFAR2 that can accurately predict mRNA secondary and tertiary structures [106]. This strategy allows for the improvement of mRNA quality without altering experimental parameters or requiring additional purification steps.

The formation of truncated byproducts caused by hydrolysis and RNase activity can be reduced by adding RNase Inhibitor into the IVT reaction and the use of RNase inactivating detergents for workspace cleaning. Metal ion contamination can be reduced by using high quality consumables, storage containers and bottles.

### Analytics for mRNA fragmentation

A common method to analyse mRNA integrity is Capillary Gel electrophoresis (CGE) [24]. This technique separates mRNA by their size under denaturing conditions, which eliminate secondary structures, using agents such as formamide or urea [24, 107].

It can efficiently separate oligonucleotides greater than 2000 nt in length and can also detect mRNA aggregates but its limitation lies in the long time of the separation which makes it less efficient for mRNA drug and process development. Recently, a study developed a microchip capillary electrophoresis (mCGE) to analyse purity and integrity of an RNA 2000 nt in length [108].

The separation time could be reduced to one minute and is suitable for analyse of various lengths of RNAs by separating an RNA mass ladder that ranged from 200 to 6000 nt.

This method is a useful approach to separate mRNAs and get information about the RNA integrity. Smaller RNA fragments like abortive transcripts (which are much shorter than 200 nt) cannot be detected with this method.

Fragmented mRNAs can also be analysed by IP-RP HPLC and radioactive labelled gel electrophoresis, as discussed in 3.4 [24, 40].

Another, more sensitive method to analyse short RNAs is Liquid-Chromatography-Mass spectrometry (LC-MS). The length of the transcripts can be measured and distinguished between single-stranded RNA, RNA aggregates, and double-stranded RNA (dsRNA) [24]. In one study it was shown that oligonucleotides as short as 4 nt can be analysed [109], making it an attractive and powerful tool for abortive transcript analysis.

## Uncapped mRNA

### Formation of uncapped mRNA

Capping of mRNA is achieved by adding a 7-methylguanosine moiety to the 5’triphosphate end (m7GpppG) of the mRNA.

**Fig. 8:**
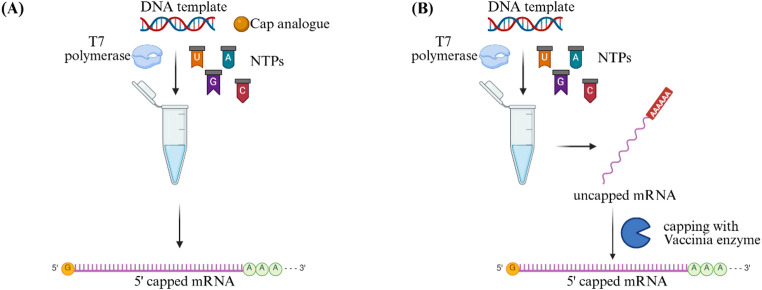
The two pathways to cap mRNA in vitro: **(A)** co-transcriptional capping during IVT reaction. All components, including DNA template, T7 RNA polymerase and NTPS are mixed with the cap analogue. **(B)** post-transcriptional capping. Immediately after IVT reaction, produced mRNA is capped by the *Vaccinia* virus capping enzyme. Created in BioRender. Nowak, C. (2025) https://BioRender.com/4fsm3w9

There are two pathways to cap mRNA in vitro: co-transcriptional using capping analogues that are directly incorporated during IVT and enzymatically after IVT (post-transcriptional) using the *Vaccinia* virus capping enzyme (Fig. 8). With the post-transcriptional pathway, the 5’ end of the mRNA, pppN, is transformed by this enzyme using guanine from GTP and S-adenosylmethionine to form the terminal cap GpppN (G cap) which is then methylated by (guanine-7)-methyltransferase activity to the N7-methylated guanosine triphosphate cap (m7GpppG). This capping structure is referred to as Cap 0 [110–114].

**Fig. 9:**
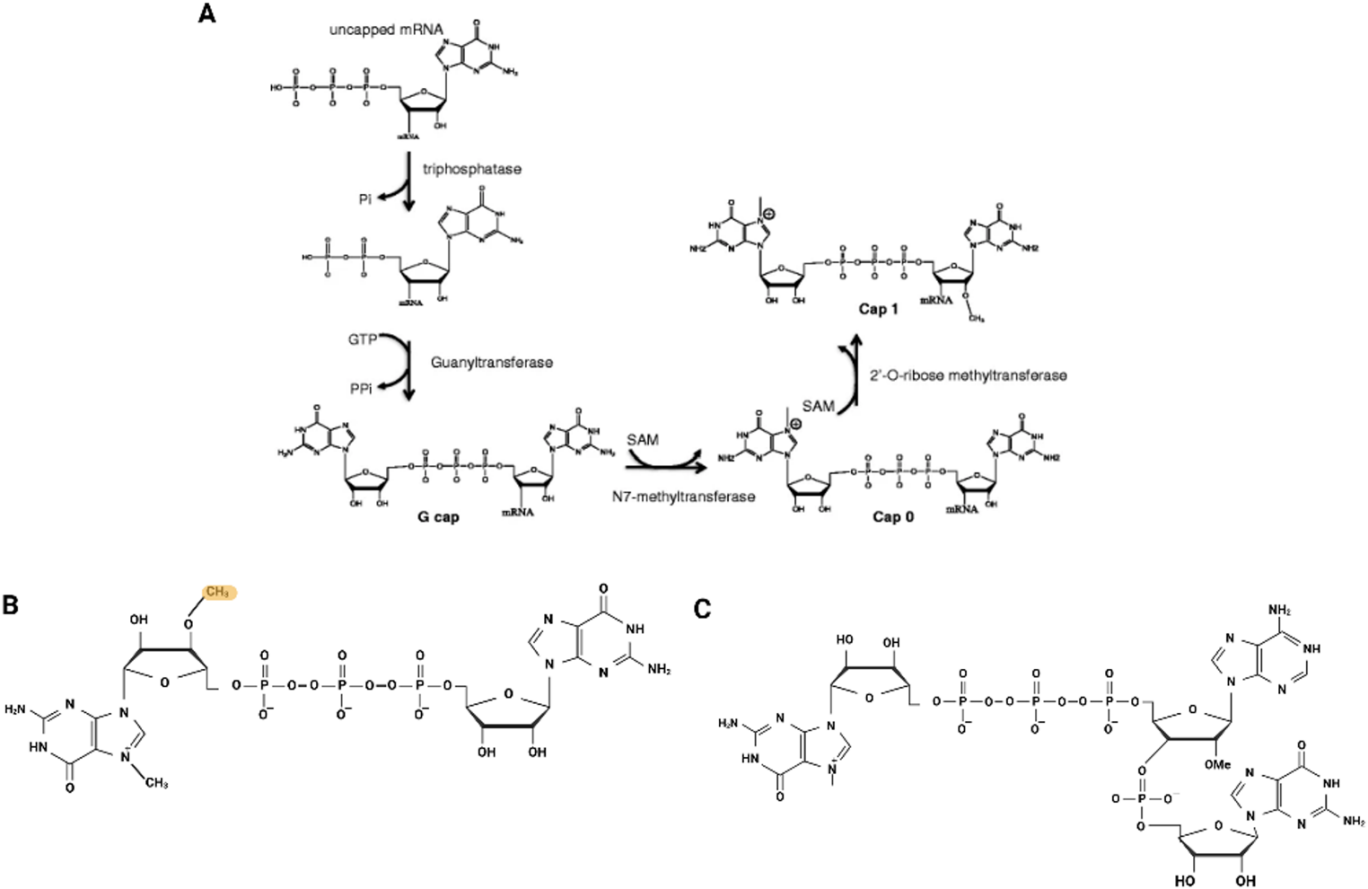
(**A**)The enzymatic capping pathway and capping structures of mRNA. S-adenosylmethionine is used as a methyl donor and the uncapped mRNA is capped first with triphosphatase activity, then guanyltransferase which forms the G cap and with N7-methyltransferase this G cap is formed to Cap 0. This can then be further modified to Cap 1 by 2’-O-ribose-methyltransferase. Reprinted and modified with permission from [112]. (**B**) Structure of ARCA capping analogue which is blocked at the 3’-hydroxyl group of the m7G, marked in yellow. (**C**) Schematic illustration of co-transcriptional capping analogue CleanCap structure (by TriLink Biotechnologies, cat no. N-7113). Created in BioRender. Nowak, C. (2026) https://BioRender.com/t9xeulw

By 2’-O-methylation, Cap 0 is formed to m7GpppmG, called the Cap 1 type (Fig. 9A) [112, 113].

During co-transcriptional capping a capping analogue such as m7GpppG is added to the IVT reaction which is then incorporated by the T7 RNA polymerase at the 5’ end instead of a GTP [112]. Beside Cap 0 and Cap 1, there are also other capping structures, Cap 2 and Cap 4 which are produced through additional 2′-O-methylation steps [115], although they are less commonly used.

During co-transcriptional capping some cap analogues can be incorporated in the reverse orientation (GpppGm7) resulting in the formation of wrong capped mRNA byproducts, lowering the translation efficiency of the mRNA. Up to 50% of co-transcriptional capping can be reverse orientated [112, 116, 117]. This is because the m7GpppG is linked by a 3’−5’ phosphodiester bond to the first nucleotide residue of the mRNA chain [117].

A competitive interaction also occurs between cap analogues and GTP during transcription, often leading to partially uncapped mRNA species. To improve capping efficiency, the GTP concentration in IVT reactions is typically reduced and cap analogue concentration increased; however, this adjustment can compromise overall mRNA yield [112].

Currently, there are three common cap analogues for co-transcriptional capping that are broadly used for the studies of therapeutic mRNA: the m7GpppG dinucleotide, the 3’-O-Me-m^7^G(5’)ppp(5’)G RNA cap analogue (ARCA) (Fig. 9B), and CleanCap a trinucleotide (Fig. 9C). ARCA is a modified capping analogue blocked at the 3’-hydroxyl group of the m7G which ensures that the cap analogue is incorporated in the correct orientation and has reported capping efficiencies up to 80% [118].

The third capping analogue is CleanCap (cap 1 structure) a trinucleotide, which forms base pairing during IVT initiation and reported to achieve > 90% capped mRNA and requires a starting sequence of “AG” [119, 120].

### Impact of uncapped mRNA

The 5’-cap structure of mRNA plays a pivotal role in maintaining its stability and enabling efficient translation, making it essential for both natural mRNA function and mRNA-based therapeutic applications [112]. It participates in several key processes, including translation initiation, mRNA splicing, nuclear export, and turnover, all of which influence the steady state levels and functional lifespan of mRNA within the cell [115, 121, 122].

mRNAs that are uncapped or possess incorrectly oriented caps are not efficiently recognized by the ribosome, resulting in poor overall translation efficiency.

Moreover, the cap structure stabilizes the mRNA by shielding it from exonucleolytic degradation, thereby enhancing its half-life and allowing for sustained protein synthesis [123–125].

Consequently, uncapped mRNA undergoes rapid degradation, compromising its stability and diminishing the therapeutic impact. Therefore, understanding and optimizing mRNA capping is fundamental to advancing effective mRNA-based therapies.

### Mitigation of uncapped mRNA

Cap analogues have been engineered to prevent incorporation in the reverse orientation during IVT, known as the anti-reverse cap analogues (ARCAs) [117]. A methyl group was included at the 3’-OH of the m7G ribose to create 3’-O-Me-m7GpppG capping analogues [126]. At this position the first dinucleotide caps are correctly incorporated during the IVT [127, 128]. This kind of modification is also used in COVID-19 mRNA vaccines [129]. Modified cap analogues with a higher affinity to the eukaryotic translation initiation factor 4E (eIF4E) have proven to increase the initiation of translation [121]. Additionally, the mRNA stability can also be improved by modifying the phosphate chain in the cap structure to make it more resistant to exonucleases that causes decapping. With this strategy, the lifetime of the mRNA in the cell can be increased [127, 130].

Among available analogues, CleanCap™ trimer has demonstrated the highest capping efficiency and provides a natural Cap 1 structure, which supports superior translation compared to the Cap 0 structure achieved with ARCA [119].

From a manufacturing perspective, co-transcriptional is preferred over post-transcriptional capping. It is less time-consuming, and the manufacturing process can get more difficult to control if enzymatic reactions are involved due to their high fail rate based on enzyme quality and conditions [131].

The most effective co-transcriptional capping reagent is CleanCap reaching the highest reported capping efficiencies. However, it is costly, making the capping process itself to the most expensive part in mRNA manufacturing.

### Analytics of uncapped mRNA

A common method to analyse uncapped mRNA is LC-MS [112]. However, LC-MS cannot analyse the full-length mRNA, therefore short sequences from the 5’-cap have to be cleaved using an enzyme or ribozyme [120, 132]. The cleaved fragment is then hybridized with an RNase H cleavage probe and can be analysed with LC-MS. Uncapped and capped mRNAs are investigated by their different masses and retention times [112, 133].

An alternative approach used to detect uncapped mRNA is to use enzymatic digestion into smaller fragments (< 50 base pairs) followed by polyacrylamide gel electrophoresis. The capped mRNA can be distinguished from un-capped mRNA by size. This is an in-expensive, semi-quantitative and simple method but includes many handling steps which lead to a higher risk for mistakes and the low throughput [133, 134].

Another effective method for assessing capping efficiency involves the selective enzymatic digestion of uncapped mRNA using a two-enzyme approach. This method, first described in 2017 by Chiron & Jais, involves the sequential use of RNA 5’polyphosphatase and terminator 5’phosphate dependent exonuclease. The 5′ polyphosphatase removes the γ and β phosphates from uncapped mRNA, converting it into a 5′ monophosphate form. This modified RNA is then specifically degraded by the exonuclease. The remaining capped transcripts can subsequently be quantified using HPLC, allowing for an accurate estimation of capping efficiency [135, 136].

Labelling the 5’-cap with a fluorescent marker by using capping analogues with a fluorescent tag or chemically modified guanosine nucleotides is another analysis technique [133, 137]. It is possible to label the phosphate with fluorescein, an organic compound [138]. It was shown that the translation is not affected by this method, however high signal background, fluorescence stability and labelling efficiency are reasons why this analytic approach is not widely used [133].

A recent approach is to detect uncapped and capped mRNA with Nanopore-sequencing, a method used for direct sequencing of RNA and its modifications [139]. In this technique, the mRNA molecules are translocated through a nanoscale protein pore, and the resulting changes in ionic current are measured to determine the sequence and structural features of the RNA [140]. This method has been improved by an engineered nanopore to directly identify various RNA modifications such as pseudouridine and N7-methylguanosin (m7G) with an accuracy of 99.6% [140]. Nanopore-sequencing has the potential to identify modifications such as cap structures and, therefore, to distinguish between capped and uncapped mRNA, but it requires further optimization. Apart from that, it is time consuming, as the samples must be clean and a library has to be prepared according to Oxford Nanopore Technologies’ instructions [141].

Another method is quantitative splinted-ligation reverse-transcriptase PCR (qSL-RT-PCR) [142]. In this method, an overhang upstream at the 5’-end of the mRNA is created by using an oligonucleotide splint. This overhang is annealed with an oligonucleotide anchor on which the uncapped mRNA can be ligated with its free 5’ monophosphate. Since capped mRNA is blocked and cannot be ligated onto the anchor only uncapped mRNA can be detected. With this method it is possible to quantify the amount of uncapped mRNA in the IVT product. This is a relatively complex method, making it unsuitable for high-throughput analysis. Only if multiplexed in a single PCR setup in which multiple targets are detected in the same reaction well or tube, this method becomes moderate high-throughput [143].

At this stage, most of the current methods are either slow, expensive, inaccurate or require further optimization and specialised equipment. The two-step enzymatic digestion combined with HPLC is the simplest and cost-effective method accessible for most laboratories. Table 2 summarizes the current analytical methods and their effectiveness.

**Table 2:** Summary of current analytical methods for capped mRNA and their suitability for industrial applications considering sensitivity, specificity and sample throughput.

Analytical method	Sensitivity	Specificity	Throughput
Two-step digestion with RP-HPLC	High	High	High
LC-MS	High	Middle - need enzymatic digestion prior analysis	High
Polyacrylamide gel electrophoresis	High	Middle - need enzymatic digestion prior analysis	Low
Fluorescent marker	Low	Low	Low
Nanopore-Sequencing	High	High	High
Multiplexed qSL-RT PCR	High	High	Medium high

## Process related impurities

Beside the product related impurities there are also process related impurities that need to be removed during purification to meet regulatory requirements [144]. The DNA template, salts, residual nucleotides that were not incorporated into the mRNA, the T7 RNA polymerase as well as endotoxin and maybe metal ions remain in the sample after the IVT reaction has stopped. For these impurities it is impossible to implement mitigation strategies as all those components are needed for a successful IVT reaction. Since these components are essential for transcription, direct mitigation is not feasible; instead, their removal relies on robust downstream purification.

### Residual DNA, RNA-DNA hybrids and RNA polymerase

The process related impurities vary in their sizes, DNA is typically the largest undesired molecule in the crude IVT reaction mixture and is commonly removed by enzymatic digestion with DNaseI, which also serves to terminate transcription [2].

This step is highly important as an exceeding amount of DNA in the vaccine would lead to severe inflammatory reactions [145]. Therefore, the amount of residual DNA recommended by the regulatory authorities is 10 ng DNA per dose [145, 146]. It has been proven, by performing a Qubit fluorometric quantitation assay and LC-MS, that in the COVID-19 vaccines from Moderna (Spikevax) and BioNTech (Comirnaty) the DNA content was below and in some samples 1.8-fold above this threshold [145]. It is worth noting that the Qubit system is not suitable for measuring mixtures of nucleic acids, as it exhibits non-specific interactions and requires enzymatic destruction of either RNA or DNA, depending on the target to be measured [147]. Another study used multiple orthogonal methods: qPCR, fluorometry, capillary electrophoresis, and sequencing with biological and technical replicates of the COVID-19 vaccines Spikevax and Comirnaty [147]. They could prove that in all samples and in all analytical methods the DNA content was consistently below the established threshold of 10 ng per dose. These findings confirmed the regulatory compliance of mRNA vaccines and the need of complementary approaches to achieve reliable vaccine analysis [147]. Another critical by-product that is being formed during the IVT process are RNA-DNA hybrids. Those are most likely also sensed by innate immune response sensors like dsRNA since several PRRs can detect them [18–20]. By performing the Dot Blot assay with the antibody S9.6 mAb those impurities can be detected in the sample [21]. DNaseI removes RNA-DNA hybrids quite inefficiently, its activity is at least 100-fold lower than for dsDNA [148]. Additionally, RNA-DNA hybrids can also not be removed by cellulose-based chromatography [21]. Therefore, developing methods for efficient and reliable removal of RNA-DNA hybrids are needed, for example by optimizing chromatography approaches.

The residual T7 RNA polymerase can be effectively removed by using several purification techniques, such as chromatography.

### Endotoxin and residual NTPs

Due to the fact that the DNA template and RNA polymerase are produced using bacterial expression systems such as *E. coli* control of microbial impurities, including viral, bacterial and endotoxin contaminants, is critical for ensuring product safety and compliance with international regulatory standards. The ICH Q5A(R2) guideline provides a framework of viral safety evaluation in biotechnological products, emphasizing testing strategies and risk assessment as well as validation for potential viral contaminants introduced through raw materials or process intermediates [149].

Characterization of expression constructs and production systems is supported by the ICH Q5B, which offers guidance on ensuring the stability and identity of recombinant materials used in upstream process [150]. *E. coli* is gram-negative and lipopolysaccharides from those, called Endotoxins, are of particular concern due to their potent immunogenicity and heat resistance. Through improperly purified reagents or contaminated labware these can enter the IVT workflow. Therefore, strict quality control, including the use of endotoxin-free materials and validated endotoxin testing is essential to meet ICH requirements and to ensure the safety of mRNA therapeutics.

Chromatography is the method of choice for efficient, large-scale purification of biopharmaceuticals and mRNA products due to selectivity and scalability.

A highly effective approach is oligo(dT) affinity chromatography. This affinity chromatography uses immobilized oligo-deoxythymidicil acid (oligo(dT)) ligand and its base-pairing with the poly(A) tail of the mRNA. Through complementary base-pairing only full length mRNA containing a poly(A) tail can bind to this ligand and impurities, such as residual NTPs and fragments remain in the flow through. Afterwards, it and can be eluted from the column leading to a high purity product with a single purification step [151].

Oligo(dT) columns cannot remove dsRNA impurities that contain a ploy(A) sequence and hence additional polishing steps are necessary [2].

One common polishing method is IP-RP-HPLC, which effectively removes dsRNA, thus, enhancing translation efficiency and reducing immunogenicity [69]. This method requires large amounts of toxic solvents such as acetonitrile and ion-pairing reagents, which must be carefully removed post-purification which poses environmental and scalability challenges.

Anion exchange chromatography (AEX) is another method for separation of mRNA from process-related impurities [2].

AEX is potentially limited in resolving mRNA product-related impurities such as fragments and dsRNA, and its full potential for mRNA manufacturing has not been fully explored yet.

To summarize, the purification of mRNA products needs further development as the currently applied methods all have limitations, and a platform process such as for monoclonal antibodies has not been developed yet.

## Conclusion and future perspectives

The success of mRNA vaccines during the COVID-19 pandemic has demonstrated the enormous potential of mRNA therapeutics. However, achieving consistent manufacturing of high-quality mRNA remains a significant challenge, primarily due to product- and process-related impurities such as dsRNA, mRNA fragments and uncapped transcripts. These impurities can elicit undesirable immune responses, reduce translation efficiency and impact the overall therapeutic outcome. Therefore, their identification, quantification, and control are critical for ensuring product safety and performance. Despite their importance, current research and regulatory efforts are not yet placing sufficient focus on impurity control. Existing guidelines for preclinical mRNA therapeutics remain vague and underdeveloped.

Recent research has provided important insights into the mechanisms of impurity formation and has proposed promising mitigation strategies, including optimization of IVT conditions, use of chemically modified nucleotides, purification of linearized DNA templates and development of engineered RNA polymerases. Analytical technologies such as IP-RP HPLC, capillary gel electrophoresis and nanopore sequencing have advanced the ability to characterize and quantify mRNA impurities; however, these techniques still face limitations related to throughput, cost, and sensitivity.

Future efforts should prioritize the development of standardized analytical methods and scalable purification platforms. Equally important is a deeper understanding of the IVT reaction to optimize conditions for both yield and product quality. Given the complexity of IVT, with multiple enzymatic components and reaction parameters, systematic studies are needed to elucidate how each factor influences the generation of mRNA fragments, dsRNA, and the kinetics of initiation and elongation. Effective impurity control begins with minimizing their formation, particularly dsRNA, during synthesis. Furthermore, advances in AI- and machine learning–driven design of DNA templates and mRNA structures offer promising avenues to intrinsically reduce impurity formation at the sequence level.

From a regulatory perspective, increased focus is needed on defining acceptable impurity thresholds and developing global standards for mRNA therapeutics, similar to established frameworks for biologics like monoclonal antibodies. Future research should prioritize developing standardized analytical methods for better in real-time process monitoring as well as scalable purification platforms. Regulatory bodies should define clearer guidelines on acceptable impurity levels, facilitating wider adoption of high-quality mRNA therapeutics. Achieving these goals will be crucial to ensure the safe, effective and widespread use of mRNA therapeutics in the future, extending their application beyond infectious diseases into areas such as oncology, rare diseases and regenerative medicine.

## Data Availability

Not applicable.

## Abbreviations

5′pp: 5′-Diphosphate end of RNA
5′ppp: 5′-Triphosphate end of RNA
AEX: Anion Exchange Chromatography
AI: Artificial Intelligence
AO: Acridine Orange
ARCA: Anti-Reverse Cap Analogue
Bp: Base pair(s)
cDNA: Complementary DNA
CGE: Capillary Gel Electrophoresis
COVID-19: Corona Virus Disease of 2019
α-^32^P-CTP: Cytidine triphosphate labelled with phosphorus 32 (^32^P) on its alpha-phosphate group
CQA(s): Critical Quality Attribute(s)
DNA: Deoxyribonucleic Acid
DNase I: Deoxyribonuclease I
dsRNA: Double-stranded RNA
E. coli: Escherichia coli
EC: Elongation Complex
eIF4E: Eukaryotic Translation Initiation Factor 4E
ELISA: Enzyme-Linked Immunosorbent Assay
FARFAR: Fragment Assembly of RNA with Full Atom Refinement
GMP: Good Manufacturing Practice
GpppGm7: Reverse-oriented cap analogue (undesired orientation)
GTP: Guanosine Triphosphate
α-^32^P-GTP: Guanosine triphosphate labelled with phosphorus 32 (^32^P) on its alpha-phosphate group
HPLC: High-Performance Liquid Chromatography
IC: Initiation Complex
ICH: International Council for Harmonisation
ICH Q5A(R2): International Council for Harmonisation Guideline on the Viral Safety Evaluation of Biotechnology Products Derived from Cell Lines and Human or Animal Origin
ICH Q5B: International Council for Harmonisation Guideline on the Analysis of the Expression Construct in Cells Used for Production of r-DNA Derived Protein Products
IFN(s): Interferon(s) (Type I/III)
IP-RP-HPLC: Ion-Pair Reverse-Phase High-Performance Liquid Chromatography
IVT: In vitro Transcription
J2 mAb: J2 monoclonal antibody (anti-dsRNA)
LC-MS: Liquid Chromatography–Mass Spectrometry
M: Molar
m5C: 5-Methylcytidine
m6A: N6-Methyladenosine
m7GpppG: 7-Methylguanosine triphosphate cap (Cap 0)
mAb: Monoclonal Antibody
mCGE: Microchip Capillary Gel Electrophoresis
MDA5: Melanoma Differentiation-Associated protein 5
Mg^2+^: Magnesium ion
MgCl₂: Magnesium Chloride
ML: Machine Learning
mRNA: Messenger RNA
NaCl: Sodium Chloride
Nt: nucleotides
NTP(s): Nucleoside Triphosphate(s)
Oligo(dT): oligo-deoxythymidicil acid
ORF: Open Reading Frame
PAGE: Polyacrylamide Gel Electrophoresis
Pb^2+^: Lead(II) ion
PCR: Polymerase Chain Reaction
PETfold: Probabilistic Evolutionary and Thermodynamic
PPi: Inorganic Pyrophosphate (diphosphate)
PRR(s): Pattern Recognition Receptor(s)
^32^P-labelled: Labelled with phosphorus 32 (^32^P)
qSL-RT-PCR: Quantitative Splinted-Ligation Reverse-Transcriptase Polymerase Chain Reaction
RIG-I: Retinoic acid-Inducible Gene I
RNA: Ribonucleic Acid
RNase: Ribonuclease
RP-HPLC: Reverse-Phase High-Performance Liquid Chromatography
SP6: SP6 RNA Polymerase (bacteriophage)
ssRNA: Single-stranded RNA
T3: T3 RNA Polymerase (bacteriophage)
T7: T7 RNA Polymerase (bacteriophage)
TEAA: Triethylammonium Acetate
UTR(s): Untranslated Region(s)
UV: Ultraviolet
VAX-seq: Vaxcine Quality Analysis using RNA sequencing
